# Memoirs of the Late Dr. Beddoes

**Published:** 1809-03

**Authors:** 


### MEMOIRS of the late Dr. BEDDOES.

IN our last we announced, on the authority of Mr. Geddy, that the public are to expect some account of the life, writings, and practice of this celebrated character, from Mr. King of Clifton, and Dr. Fox of Bristol. In the mean while, our gratitude to so valuable a contributor, as well as our wish to meet the public impatience, induce us to offer the following particulars from a source, the authenticity of which cannot be questioned.

---

THOMAS BEDDOES was born at Shifnal, in Shropshire, about the year 1754 or 1755. His relations were respectable and opulent people, nearly all whom were engaged in trade. The father was a tanner, but seems to have been determined in early life that the son should receive an excellent education, so as to be fitted for a higher sphere in society. Accordingly, after obtaining that species of knowledge usually procured in the provincial schools, the distant prospect of Oxford terminated the visto of his classical prospects.

In consequence of the laudable ambition of his friends, he was sent thither; and there is still a report extant at this university, that the settlement of the young Tyro was wholly entrusted to the care of an uncle. On entering the grand mart of learning, with which, as well as its inhabitants, he was utterly unacquainted, he instantly presented himself, along with Thomas, at the gate of St. John's; and ringing the bell, asked, "If there was any good education to be had there?" The porter, perceiving perhaps the actual situation of affairs with a single glance of his eye, like a prudent man, introduced them to the Master, and the usual fees being paid, the young student's name was actually registered on the books.

But the adventure did not conclude here; for the master, struck with the novelty of the circumstance, kept them both to dinner, when, in the course of conversation, it came out that the two strangers were provided with letters of recommendation to Dr. Surgrove, master of Pembroke, and that the uncle had imagined there was but one college in the university. On this, the money was returned with great politeness and liberality, and young Mr. Beddoes matriculated in due form at Pembroke, according to his original destination.

Of the exact year when this occurred we cannot speak with any degree of certainty, but suppose it to have been in 1778 or 1779. Certain it is, that on the 19th of July, 1783, he proceeded master of arts, and on the 13th of December, 1786, obtained the degrees of B. and M. D.

As it has been generally supposed, that a modern medical education is incomplete without a visit to Scotland, Dr. Beddoes accordingly repaired to Edinburgh, about the year 1781, or 1782, in pursuit of those liberal attainments, by which both himself and the public were afterwards to profit; for he was eminently replete with zeal, and never wished to do or to learn any thing by halves. While there, he attended the lectures of the most famous professors of the day, was noticed as a youth of great promise, and, if we are not greatly misinformed, lived in intimacy with the celebrated Dr. Brown, whose new system for a while seemed to bear down every thing before it. Sir James Macintosh, who was also intended to be a physician, and actually actually took a degree for that purpose, was one of his cotemporaries and friends.

It does not appear, however, that the subject of this memoir, at a more mature period of his life, considered the system then prevalent in North Britain as incapable of being amended; for we find him, but the year before his death, while treating of the melioration of his favourite science, expressing himself as follows:

"However the pupils of Edinburgh may succeed in the world, and fair as it may be for an advocate to avail himself of the fact, I doubt exceedingly whether the public would, if called upon to act with deliberation, yield its confidence to one of their three years' graduates. In case, for instance, of an election to an hospital, would not the shortness of his standing, and the necessary immaturity of his experience, operate as a fatal objection? Well then? if he is not fit to have pauper patients committed to him, why should others be allowed to commit themselves? It may be said, that a five or six years' graduate would be thought equally incapable of the charge. I believe quite the contrary, provided the electors should have both information and integrity enough to vote according to the merits.

"It always seems invidious, and in many cases is arrogant in an individual to adduce his opinion of a public body in argument; but as the merits of the Edinburgh school are opposed in this manner to the projected improvement of medical education, those who take a part in the question, seem called upon to declare themselves, if they have any probable cause of knowledge.

"Let me, therefore, briefly state, that I went to Edinburgh as an Oxford bachelor of arts, passed there three winters and one summer, was perpetually at the lectures of the professors, and in the societies of the students. You may think it probable that I have no humiliating associations connected with Edinburgh, if I add that I can never hope to be of so much consequence among my equals any where else, since the students heaped upon me all those distinctions which you know it is in their power to confer. Few individuals, certainly, have ever had a better opportunity of knowing any school. I have seen other schools of medicine, conversed and corresponded much, from that time to the present, with pupils and professors, studied their methods and the productions as well of the youth as of the seniors. So that I cannot accuse myself of having omitted any thing by which I might be enabled enabled to form an opinion concerning this grand question of medical instruction.

"After comparing, on the spot, the means with the end, I certainly did conceive that a more deliberate process would be preferable, and that a method of instruction, in some other respects materially different, would form physicians far more trustworthy. This opinion, various members of the medical societies could, I dare say, testify that I expressed; and every thing that I have since seen of practice and of literature has tended to confirm it. After a lapse of years, and without the smallest communication, it is satisfactory to find the associated faculty and their correspondents concurring to make it the basis of a legislative measure, and certainly without being actuated by the least ill-will towards any medical school in the universe.

"I know not whether any impartial person, after seriously reflecting upon the surest way of advancing in so difficult a study, ever surveyed the medical classes at Edinburgh. He would see that perpetual bodily hurry which is generally attended with a good deal of confusion of mind. No sooner does the college hour-bell toll, than the audience rush out in full stream, leaving the last word half finished in the mouth of one professor, not a few fearing lest they should miss the first words of another. Will you call this mere juvenile ardour? The young men there were generally, and doubtless still are, earnest in their pursuits: but it was a common feeling, that each attempted too much at once; and if it be true, that figures and hues which are to last, must be laid again and again on the mind, with pauses between to allow them to fix, somewhat as in fresco painting, this feeling would appear to be right. A calculation had been made, and the required attendance distributed as well as possible through the three years. Considering the number of professors, and the necessity for those, who were to trust to this school solely, to attend certain courses, (as the anatomical, practical, and clinical,) two or three times; considering, besides, that the merit of *out-lecturers* will have claims upon the inquisitive, and that many had no other chance for acquiring a smattering of natural philosophy and natural history, how could any student, and especially the most ardent, avoid attempting too much at once? The consequence was too apparent. Our academical architects, in their hurry to finish the structure, failed to lay a solid foundation."

It

It appears evident, that Dr. Beddoes' residence in Scotland did not prevent him from keeping his terms, and participating in the honours of his own university; for on his return, he again resorted to Pembroke, and took his degrees, in the manner and at the times already specified.

It may be necessary to state here, that chemistry had always been a favourite study with the subject of this article; and that after having first viewed it, merely as a branch of medicine, he afterwards addicted himself to this pursuit, with a more than ordinary degree of avidity. His reputation, indeed, as well as his acquirements, in this very elegant and very useful department of human knowledge, must have been very extensive, for in 1786, we find him acting as reader of chemistry to his "Alma Mater:" there was no professorship of this kind established at that period, or indeed until 1803, at Oxford, although one had been founded so early as 1706, at Cambridge.

In the course of 1787, he visited France, and appears to have been for some time resident at Dijon. While at Paris, he of course became acquainted with Lavoisier, whose reputation was, at that period, at its height, and not only acquired his esteem, but also carried on a scientific correspondence with him after his return. At the evening parties of the amiable and accomplished Madame Lavoisier, his wife, he also saw some of the first company in the French metropolis, among whom were many who have since figured in the political stage, and been swept away by the volcano that soon after burst forth. Here too, he beheld the first symptoms of that Revolution, which, after shaking France to her centre, was destined to convulse the whole world.

That an ingenious young man, who with a liberal education had imbibed generous notions of both science and government, should be disgusted with the tyranny of the Bourbons, and the horrors of an arbitrary government, even while administered under its mildest forms, by a weak but amiable prince, is little to be wondered at. He certainly, like thousands, did experience great joy at the glorious prospect, which has since been so completely blasted; and who can blame him for witnessing with satisfaction, the first efforts of the French nation; who, in 1788, and 1789, in imitation of the English people in 1688, attempted a melioration of their political system.

With ideas, such as, or at least similar to these, the mind of Dr. Beddoes became deeply imbued, and it cannot be denied, that they had a considerable effect fect on his future fortunes, studies, and pursuits. In all governments whatsoever, the idea of a reform sounds terrible to those who profit by the corrupt practices that decorate and disfigure the ancient system; and one abuse, as we know by experience, is well calculated to prop and support another. Many, therefore, who admired the talents of Dr. Beddoes, were alarmed at his principles; and in the very bosom of that University, amidst those academic groves, where the noblest, the purest, and the most enlightened principles ought to be cherished, he was doomed at one critical period to experience all the rancour of malignity, and encounter all the suspicion incident to little and contracted minds.

Towards the latter end of 1792, he voluntarily resigned his readership, of which he had been in possession for about six years, and was succeeded by Robert Bourn, M. D. It was now time for him to settle in life; but a considerable period elapsed before he could finally determine on so important an object. His eye was naturally fixed at first on the metropolis, as presenting an ample field for a man ambitious of fame, and addicted to the pursuit of science. But he soon perceived, that all the important stations were already occupied; and that for years, he could only aspire to a secondary rank among the eminent practitioners of the capital.

On this, he pitched on Bristol, where, in consequence of the vicinity of the hot-wells, which still continue to attract some of the first families in the kingdom, and the swarm of rich citizens, settled both in the town and its neighbourhood, there appeared to be full scope for an honourable and successful career.

He had not been long resident there, when the prevalent disease of consumption, to palliate which the exercise of his professional talents was so often invoked, engaged his particular attention. Calling in chemistry to the assistance of medicine, he formed a notion that it was possible to cure this cruel disorder, by changing the medium which the patients respired, and this gave birth to the Pneumatic Institution, established by him. As the attempt was founded on general benefit, and the fortune of a single individual could not be sacrificed with any degree of prudence to such an undertaking, many noblemen and gentlemen, we believe, and among others the late Marquis of Lansdowne, entered into a subscription to enable him to defray the expence. Of the success, I cannot speak with any degree of certainty, and am upon the the whole inclined to consider the experiment as more curious than useful. It was, however, attended with one effect, that has in the end proved highly favourable, as well as eminently beneficial to science; for it was the means of introducing Mr. Davy to public notice, that gentleman having assisted Dr. Beddoes in constructing the apparatus, and performing the various experiments, during the course of six months.* To the honour of both parties, although they separated at the end of this period, yet they preserved an unbroken friendship, and an uninterrupted correspondence, with each other, until death snatched the pen out of the hands of one of them, and put an end to a connexion, founded on mutual regard.

I shall now endeavour in this place, to take a survey of the literary life and labours of Dr. Beddoes, without any particular attention either to dates or subject.

It is pretty evident, that for some time at least, he attempted, like the celebrated Dr. J. Jebb, occasionally to unite politics with medicine; and while acting as a physician, resolved not to omit those duties which appertained to him as a man. We accordingly find him attending a committee, which had been convoked preparatory to a general meeting of the inhabitants of Bristol, during the progress of Mr. Pitt, and Lord Grenville's "restrictive bills." Soon after this, (1796) appeared an "Essay on the public Merits of Mr. Pitt," by THOMAS BEDDOES, M.D. printed for Joseph Johnson, St. Paul's Church Yard. It is dedicated as follows:—

"To the House of Commons,
An Assembly
Whose Acts for the late Twenty Years,
No Man
Who feels for
Asia, Africa, America,
Or Europe,
Can regard,
Without the profoundest emotions."

As an introductory motto to Chap. i. we find the following couplet:

"Penned be each pig within his proper stye;
Nor into state concerns let Doctors pry."

In

* An account of the life and scientific labours of Mr. Davy, will be found in the "Public Characters for 1809."

In the course of this pamphlet, the author gives a sketch of the administration of Lord North and Mr. Pitt. The attachment of the nation to the latter of these, is attributed, 1. To his name. 2. To his "high-flying" speeches on the popular topics of influence and corruption. 3. "In virtue of his youth, he gained credit for incorruptible integrity." 4. His manner was advantageous; he declaimed pompously, and when he reasoned, he gave proofs of a quick, discerning, and cultivated mind. His speeches, in relation to his age, deserved distinguished approbation; they obtained blind admiration. An hundred young men at school and college would, in an essay, have turned the common places on liberty and patriotism, with equal dexterity, against the discomfited conductors of the American war. But not one could have been so trained in the habit of uttering them promptly. Fluency of elocution, however, does not appear to be more closely connected with wisdom, than facility or elegance of composition. 5. "By an act (the refusal of the office of clerk of the Pells in Ireland,) which as it might equally proceed from patriotic disinterestedness and the lowest cunning, his future conduct could alone render unequivocal, he confirmed the faith of a credulous people." 6. "Certain candidates for power incurred our displeasure, and we, cool, dispassionate Englishmen, took their rival to our bosom in pure despite."

In another part of this pamphlet, he exclaims, "O! superstitious nation! to whom an idol is necessary, though with the simple African thou be reduced to worship a serpent, or a crocodile with the stupid Egyptian!" And soon after he adds: "it is moderate to assert, that neither Scipio, when he had delivered Rome from her most formidable rival; nor Washington, the founder of American independence, received more enthusiastic adoration than the political adventurer, whose patriotism rested on the same blustering evidence as Bobadil's valour."

In 1802, appeared "Hygëia, or Essays, Moral and Medical, on the Causes Affecting the Personal State of the Middling and Affluent Classes". This work, which was printed at Bristol, consists of three volumes, and contains a variety of papers on personal prudence, and prejudices respecting health; on personal imprudence; British characteristics; on the use of tea; exercise; cloathing; schools; infancy; a more advanced age; catarrh; scrophulous constitution; consumption; liver complaints; plaints; gout; disorders, called nervous; febrile contagious diseases, &c. &c.

In 1803, he published " A Letter to the Right Honourable Sir Joseph Banks, K. B. P. R. S. On the Causes and the Removal of the Prevailing Discontents, Imperfections, and Abuses, in Medicine," with the following motto: Take Physic, Physic." On this occasion, he appears to join in the " hue and cry raised against incompetent possessors of diplomas," and affects somewhat of that superiority over the M. D's of the Scottish metropolis, which they themselves are said to evince, " while looking down on the sons of Aberdeen and St. Andrews, with as much pride as was felt by Mars, when he was seated at the right hand of Jupiter."

Of this spirited, ingenious, and in our opinion impartial work, we have already given a detail in the critical part of our Journal.

It is well known that the excessive heat of last summer, particularly during the harvest season, proved fatal to many of the husbandmen, particularly in the county of Glocester. Dr. Beddoes did not lose this favourable opportunity of expatiating on those virtues of sobriety and order, the want of which is so destructive to the lives of the sturdy British peasantry. He proved with much facility, that whilst they expended so much in drink, it was to little purpose they laboured, to gain merely to spend, not only their pecuniary gainings, but their very existence.

In August 1808, he transmitted two cases of hydrophobia, which were inserted in the " Medical and Physical Journal," for September. We need hardly add how much we were indebted to him at other times; and above all, how rich his communications were in some of our latest Numbers, and how valuable from the dissections which accompanied them. His other literary labours are too numerous for us to offer even title pages; doubtless, they will be accurately remembered in the promised publication.

We must here conclude the life and literary career of this extraordinary man, at the same time. The physician whose mind was ever on the stretch, to extend the confines of medical science, and discover efficacious remedies for the relief of others, at last became a patient himself. He had for some time anterior to his death, exhibited manifest symptoms of dropsy, but never considered his end as so near. His dissolution perhaps was hastened by the rigour of the present winter, for he complained frequently of cold at his extremities, and had actually sent to London

London for an ingenious mechanic, who had undertaken to warm his apartment to an equable temperature by the means of steam. His death occurred on the 24th of December, 1808; and on being opened, it was clearly discernible that the machinery had been worn out, and that the animal functions were necessarily suspended from the progress of disease. The left lobe of the lungs was found to be in a morbid state, and, as might have been easily predicted, a lodgement of water had also been effected.

Thus died, after he had attained the fifty-second, or fifty-third year of his life, Thomas Beddoes, a man who possessed a warmth, a zeal, an ardour for the pursuit of medical science, which had seldom been equalled by any, and was assuredly excelled by none. His whole life was devoted to experiment, to enquiry, to correspondence with men of talents, and to the instruction of himself and others. He possessed a fine genius for poetry, and had the happy faculty of viewing every subject on its most brilliant side. His language was glowing, figurative, and sometimes even sublime. He despised quackery, and pretensions of every kind; and was accustomed to detect and expose these to the full as freely in his own as in other professions.

In all the social relations of life, his conduct uniformly bore testimony to the excellence of his heart; for he was a good friend, a good father, and a good husband. A few years since, he married Miss Edgeworth, a lady of a respectable literary family in Ireland, by whom he has left four children.

Further particulars of his life will be speedily published under the auspices of his friends:—a work, which, if written with ability, cannot fail to be productive both of amusement and instruction.

It is to be hoped, a portrait of Dr. B. has been in some way obtained, for it was one of his peculiarities, to refuse the frequent solicitations of some of his best friends to sit for his picture.

